# Heterogeneity of ERG expression in prostate cancer: a large section mapping study of entire prostatectomy specimens from 125 patients

**DOI:** 10.1186/s12885-016-2674-6

**Published:** 2016-08-17

**Authors:** Maria-Christina Tsourlakis, Annegret Stender, Alexander Quaas, Martina Kluth, Corinna Wittmer, Alexander Haese, Markus Graefen, Stefan Steurer, Ronald Simon, Jan Korbel, Joachim Weischenfeldt, Hartwig Huland, Guido Sauter, Thorsten Schlomm, Sarah Minner

**Affiliations:** 1 Institute of Pathology, University Medical Center Hamburg-Eppendorf, Martinistrasse 52, 20246 Hamburg, Germany; 2 Martini-Clinic Prostate Cancer Center, University Medical Center Hamburg-Eppendorf, Hamburg, Germany; 3 Genome Biology Unit, European Molecular Biology Laboratory (EMBL), Heidelberg, Germany; 4 Department of Urology, University Medical Center Hamburg-Eppendorf, Hamburg, Germany

**Keywords:** Prostate cancer, ERG, Heterogeneity

## Abstract

**Background:**

TMPRSS2:ERG fusions are frequent in prostate cancer, and occur predominantly in young patients. Several studies had proposed intratumoral heterogeneity of these fusions. This study was designed to determine frequency and extent of ERG fusion heterogeneity in early-onset prostate cancer (EO-PCA, <50 years) and in elderly patients.

**Methods:**

The prostates from 63 EO-PCA and 62 elderly prostate cancer patients were thoroughly reviewed for presence of cancer foci. All 1592 tumor-containing sections were analyzed by immunohistochemistry for ERG expression.

**Results:**

The prostates included in this study contained one tumor focus in 44, two tumor foci in 21, three tumor foci in 32, four tumor foci in 15, and five or more tumor foci in 13 patients. Among 59 cancer foci with ≤3 mm, 19 (32.2 %) were homogeneously ERG positive, 39 66.1 %) were homogeneously ERG negative, and one case (1.7 %) showed a heterogeneous ERG status. The fraction of homogeneously ERG positive cancer foci remained largely constant (14–37 %) with increasing tumor focus diameter but the fraction of heterogeneous ERG findings continuously increased with tumor size and reached 39 % in cancer foci larger than 22 mm. On a patient level, ERG expression was markedly more frequent in EO-PCA than in elderly patients: 13 % of EO-PCA were homogeneously and 62 % were heterogeneously ERG positive. In elderly patients, 3 % of cancers were homogeneously and 57 % were heterogeneously ERG positive (*p* = 0.0721).

**Conclusion:**

These data show that about 20–30 % of prostate cancer foci have early ERG fusions. ERG fusions further occur in about 50 % of initially ERG negative cancer foci during cancer progression. The vast majority of cancers are heterogeneous for TMPRSS2:ERG fusions on a patient level, challenging the concept of classifying prostate cancer patients into “fusion type” and “non-fusion type” prostate cancer.

## Background

Prostate cancer is the most frequent cancer in men and represents a major cause of cancer-related mortality and morbidity [1]. Although the majority of these tumors behave in an indolent manner, a significant subset forms highly aggressive and life threatening cancers [2]. Effective curative therapies for patients with such highly malignant cancers are still lacking. The most important objectives of current prostate cancer research thus include the development of improved tools for early detection of the disease, with markers for reliably pre-therapeutic distinction between patients requiring aggressive treatment and those who do not, as well as improved systemic treatment options for patients with aggressive and metastatic disease. It is hoped, that the rapidly increasing knowledge of the molecular basis of prostate cancer will eventually lead to relevant clinical applications.

Genomic rearrangements leading to gene fusions between androgen-regulated genes and ETS transcription factors represent the most common genetic alteration in prostate cancer. The most prevalent fusion, accounting for more than 90 % of these rearrangements, links the androgen receptor (AR) responsive promoter of the TMPRSS2 serine protease to the transcription factor ERG, either by translocation or by deletion of a 3.7 megabases (Mb) segment separating the two genes on chromosome 21q22 [3]. Consequently, ERG becomes androgen regulated and is massively overexpressed in prostatic epithelium. Detecting ERG expression by immunohistochemistry and visualization of ERG rearrangements by fluorescence in situ hybridization (FISH) have proven as equally reliable methods for detecting TMPRSS2:ERG fusions [4, 5].

Based on the high frequency of TMPRSS2:ERG fusions and the potentially high impact on prostate cells by rendering ERG dependent genes androgen regulated, attempts were made to molecularly classify prostate cancer as “fusion-type” and “non fusion-type”. Several studies investigated the clinical and molecular characteristics of fusion type versus non-fusion type prostate cancer. They reported that TMPRSS2:ERG fusions occur in about 50 % of cancers and that they are unrelated to PSA recurrence in patients treated by radical prostatectomy [5] while it is possible, that fusion positive cancers might react better to anti-androgen therapy than fusion negative tumors [6, 7]. More recently, we had demonstrated, that ERG fusions occur markedly more often in young than in elderly prostate cancer patients [8].

The concept of distinguishing two clear-cut prostate cancer categories defined by presence or absence of ETS-gene-fusions has recently been challenged by reports suggesting considerable heterogeneity of ERG fusions in prostate cancer. Several studies by us [9] and others [10–16] have demonstrated ERG interfocal heterogeneity in 28–72 % of ERG-positive prostate cancers, and some of these also intrafocal heterogeneity in 4–42 % of ERG-positive tumor foci [9, 10, 12, 16, 17]. However, all these studies had suffered from some methodological insufficiencies such as a limited number of selected tissue blocks per patient, small numbers of patients, or were based on tissue microarrays, a method that only involves small tissue samples per patient [18]. To fully understand the extent of ERG heterogeneity in prostate cancer of young and old patients, we took a “brute force” effort and analyzed all 1592 tumor-containing blocks of 125 prostate cancer patients. The data reveal a very high rate of ERG heterogeneity in prostate cancer patients.

## Methods

### Patients

Sixty-three prostate cancer patients were randomly selected out of 273 patients that were treated by radical prostatectomy for prostate cancer at the age of 50 or younger (early-onset prostate cancer: EOPCA). Sixty-two additional prostate cancers from patients older than 50 years complemented the series. None of the patients received therapy prior to surgery. Cancers from old patients were matched for Gleason grade and pT stage to be comparable to the set of young patients. The patient characteristics of both groups are given in Table 1. All prostatectomy specimens were completely paraffin embedded and processed totally according to a modified Stanford protocol [19] as previously described [20]. In brief, the prostates were fixed in 37 % formalin, serially blocked at 3 mm intervals in transverse planes perpendicular to the rectal surface, and embedded in paraffin. The average number of tumor containing blocks per cancer was 13.2 (±7.8; range: 4–44).

**Table 1:** Characteristics of the 125 analyzed prostate cancers

		≤50 year	>50 year
		*n* = 63	*n* = 62
Age (yrs)	mean ± sd	44.8 ± 2.6	75.2 ± 1.5
Prostate volume (ml)	mean ± sd	28.4 ± 11.6	46.3 ± 24.5
Tumor volume (ml)	mean ± sd	3.5 ± 6.9	7.0 ± 9.9
pT stage	pT2	51	35
	pT3a	6	18
	pT3b	6	9
Gleason score	≤3 + 3	14	14
	3 + 4	34	29
	4 + 3	11	14
	≥4 + 4	4	5
Nodal stage	pN0	38	37
	pN1	4	7
	pNx	21	18
Resection margin status	R0	47	43
	R1	9	17
	Rx	7	2
Number of tumor foci	mean ± sd	2.4 ± 1.3	2.6 ± 1.7

### Histology review

**Fig. 1:**
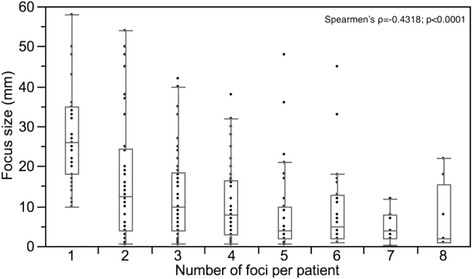
Distribution of the tumor focus size (mm) in patients in unifocal (1 focus per patient, *n* = 44) and multifocal cancers (≥2 foci per patient, *n* = 273)

For each cancer all slides were reviewed and all cancer containing sections were selected for further analysis by immunohistochemistry. For each cancer, independent tumor foci were defined according to Wise et al. [21]. In brief, tumor areas were defined as part of a single focus if they were within 3 mm of each other in any section *or* within 4 mm on adjacent sections. This method identified 1–8 independent tumor foci in our prostate cancers (mean: 2.5). Forty-four prostates had one tumor focus, 21 prostates had two tumor foci, 32 prostates had three tumor foci, 15 prostates had four, seven prostates had five tumor foci, and six prostates had 6–8 foci. For each tumor focus, diameter and Gleason score was defined. The size distribution of the individual tumor foci in patients with uni- and multifocal cancers is given in Fig. 1. In addition, individual Gleason scores were determined for the different cancer components found in the entire prostate cancer mass.

### ERG immunohistochemistry

Freshly cut sections were immunostained from each tumor containing tissue block. The antibody ERG (clone EPR3864, dilution 1:450, Epitomics) was used for ERG protein detection. Slides were deparaffinized and exposed to heat induced antigen retrieval for 5 min in an autoclave at 121 °C at pH7.8. Bound primary antibody was visualized using the EnVision™ Kit. This immunohistochemistry (IHC) protocol was previously validated against the TMPRSS2-ERG fusion status determined by FISH in a series of 453 patients. ERG rearrangement had been identified in 230 of 247 immunohistochemically ERG positive cases (93 %) but in only 2 of 206 cases (1 %) that were found negative by IHC [5].

### Interpretation of ERG immunostaining

Tumor areas were considered ERG positive, if unequivocal nuclear ERG staining was present. Negative or weak staining was validated by TMPRSS2-ERG FISH if admixed lymphocytes and/or blood vessels did not show strong ERG immunostaining. ERG immunostaining results including the percentages of positively and negatively stained areas were recorded for each individual cancer focus as well as for the entire cancer mass. At the same time staining results in PIN and non-neoplastic epithelial cells were also recorded if seen on the selected tissue slides. If ERG immunostaining was seen in tissues that did not appear to be neoplastic based on histology, AMACR and 34BE12 immunostaining was performed to either support or invalidate our histologic interpretation of normal, PIN or cancer glands.

### Statistics

The relationship between the number of tumor foci per patient and the focus size was estimated using the Spearman rank correlation analysis. Chi^2^ test was applied to test the associations between patient age and ERG heterogeneity.

## Results

### Association between tumor focus size and number of foci per patient

The size distribution of the individual tumor foci in patients with uni- and multifocal cancers is given in Fig. 1. The size of tumor foci decreased with the number of foci per patient (Spearmen’s ρ = −0.4318, *p* < 0.0001).

### ERG immunostaining at the patient level

**Fig. 2:**
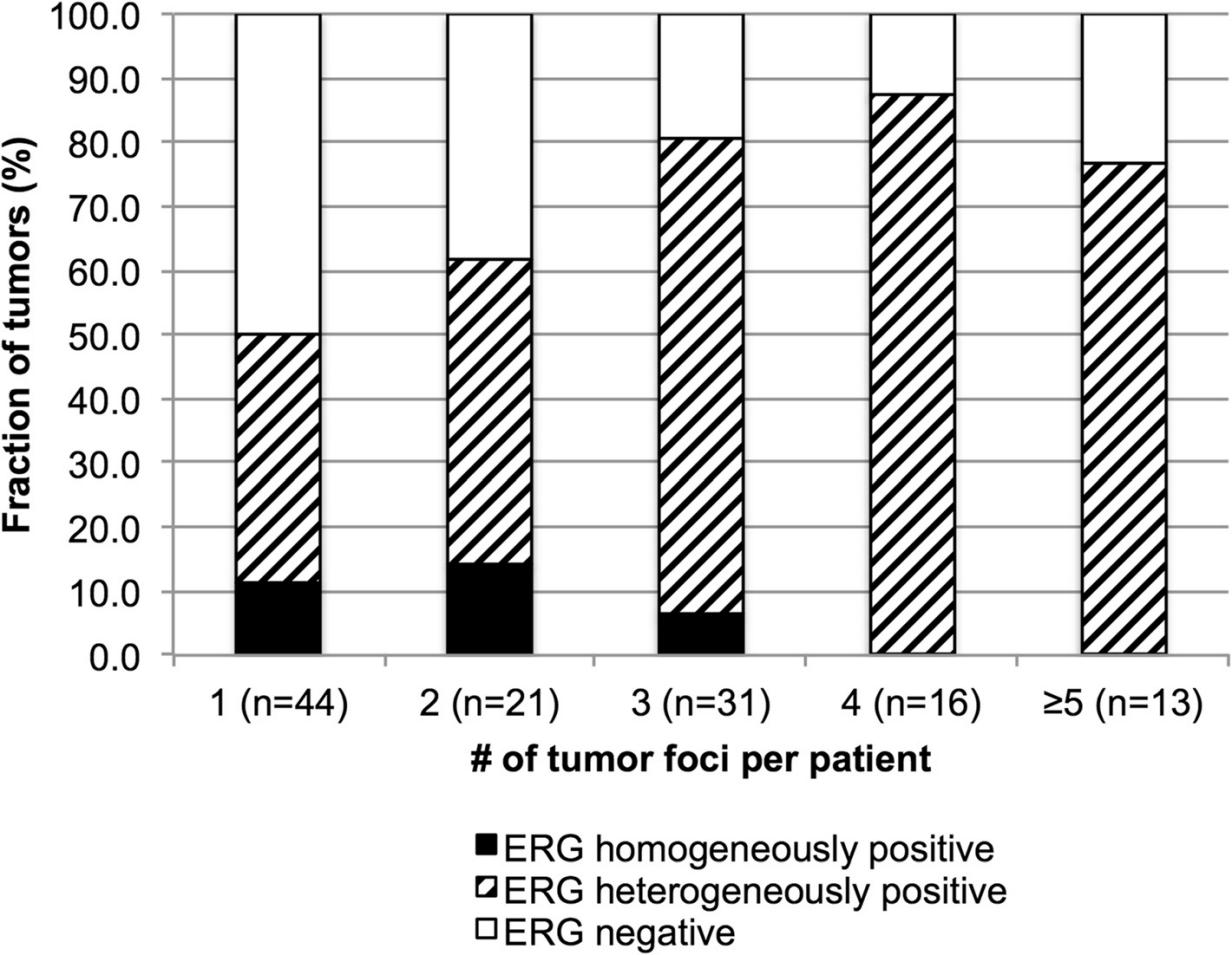
Association between the number of tumor foci and the level of ERG heteogeneity (*p* = 0.0238) on a patient basis

The prostates of the 125 patients contained 317 individual tumor foci measuring between 0.2 and 58 mm (average 13.6 mm). A patient was considered heterogeneous for ERG immunostaining if different tumor foci had different ERG results (interfocal heterogeneity) or if at least one tumor focus showed a mixture of ERG positive and ERG negative tumor cells (intrafocal heterogeneity). On a patient level, ERG immunostaining resulted in ten patients with homogeneous ERG positivity (8 %), 41 patients with homogeneous ERG negativity (33 %) and 74 (59 %) patients with heterogeneous ERG findings. Among 74 patients with heterogeneous ERG findings, there were 25 patients (34 %) where heterogeneity was only seen between different tumor foci (interfocal heterogeneity) and 49 patients (66 %) where heterogeneity was also (or only) within one or several tumor foci (intrafocal heterogeneity). It is not surprising, that the frequency of heterogeneity (on a patient basis) increased with the number of tumor foci present in a patient’s prostate (*p* = 0.0238, Fig. 2).

### ERG immunostaining at the tumor level

**Fig. 3:**
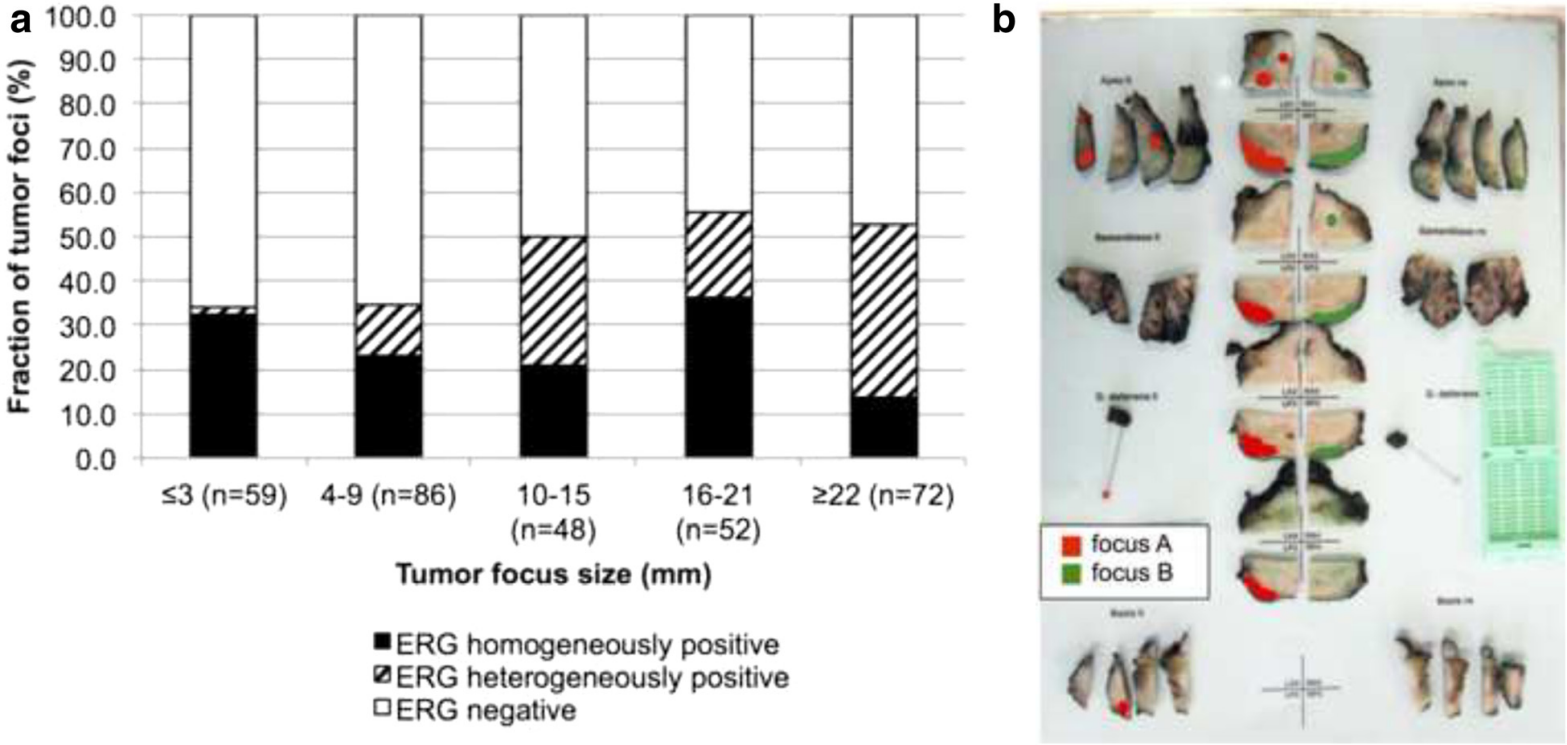
ERG heterogeneity in prostatectomies. **a** Association between the tumor focus size and the level of ERG heterogeneity (p<0.0001). **b** Example of a prostate with two separate tumor foci marked in red and green color

**Fig. 4:**
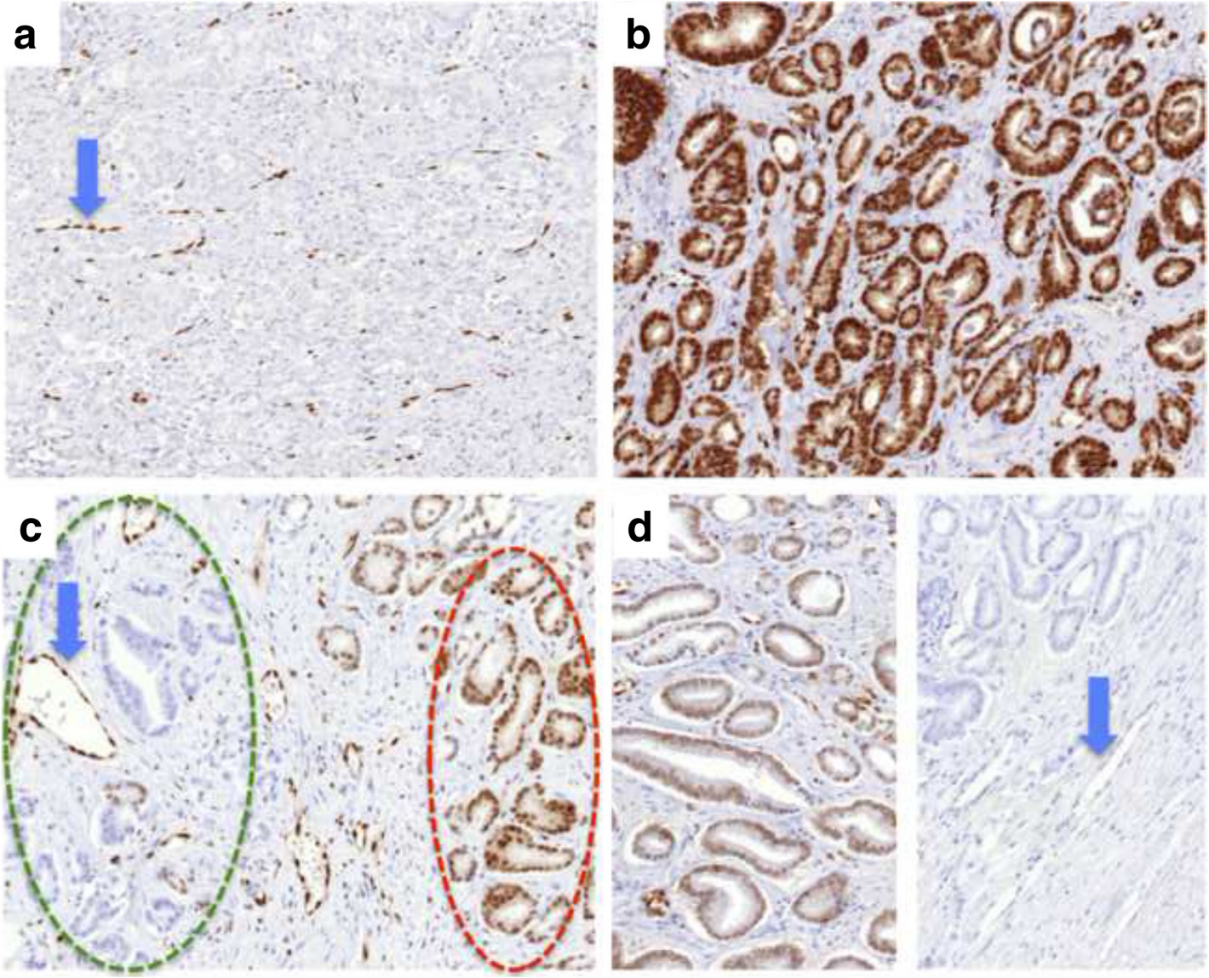
Representative images of ERG immunostainings. **a** Negative ERG immunostaining from a homogeneous ERG negative prostate cancer. The *blue arrow* indicates positive ERG immunostaining in endothelial cells as a positive control, **b** positive ERG immunostaining from a homogeneous ERG positive prostate cancer, **c** positive ERG immunostaining (*red circle*) and negative ERG immunostaining (*green circle*) from an intrafocal heterogeneous prostate cancer; the *blue arrow* indicates positive ERG immunostaining in endothelial cells as a positive control, **d** false heterogeneity, positive cancer (*left*), false negative cancer (*right*), the *blue arrow* indicates endothelial cells also lacking ERG immunostaining (**d**)

Among the 317 tumor foci identified in our cancers, 78 were homogeneously ERG positive (25 %), 176 were homogeneously ERG negative (55 %), and 63 showed a (intrafocal) heterogeneous ERG result (20 %). The fraction of heterogeneous cancers increased markedly with the size of tumor foci, while the fraction of homogeneously negative cancers decreased accordingly (*p* < 0.0001, Fig. 3). Within the 141 ERG-positive cancer foci, the majority (55.3 %) was homogeneously ERG positive, whereas 44.7 % showed heterogeneous staining, suggesting that ERG fusion often occurs only as a secondary event after tumor formation. A detectable difference in cancer morphology (i.e., gland size, gland architecture, gland density and tumor cell morphology) was not seen between ERG positive and negative cancer foci. Representative images of ERG immunostainings are given in Fig. 4.

### Relationship of ERG heterogeneity with Gleason grade

**Fig. 5:**
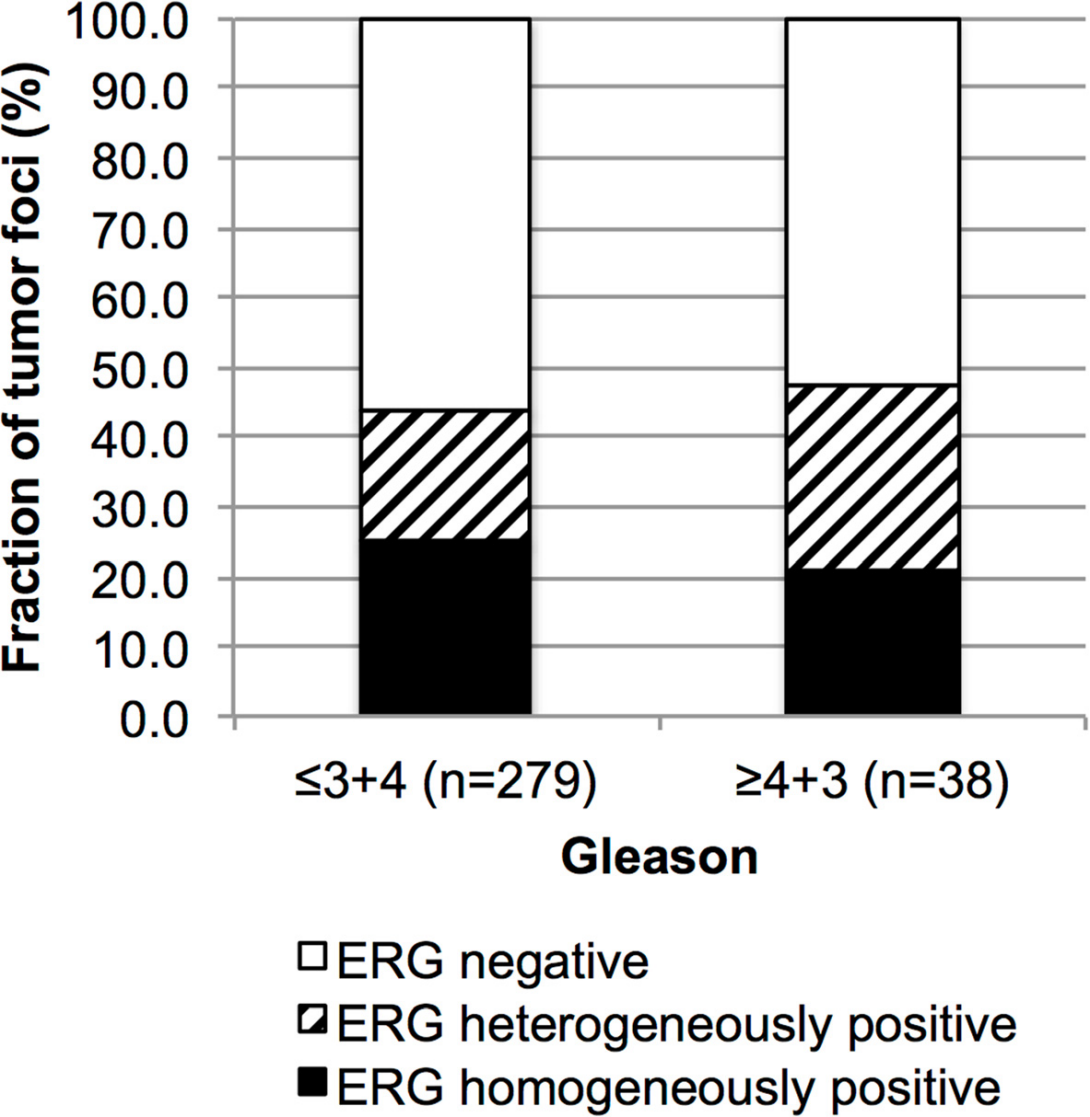
Association between the Gleason grade and the level of ERG heterogeneity (*p* = 0.5694) on a tumor focus basis

To evaluate the role of ERG rearrangements with tumor progression, we next analyzed ERG heterogeneity in tumor foci of different Gleason grades. Intrafocal ERG heterogeneity was found in 26 % high-grade cancers and in 19 % of low-grade tumors, but the difference was not statistically significant (*p* = 0.5694, Fig. 5).

### Relationship between ERG heterogeneity and patient age

**Fig. 6:**
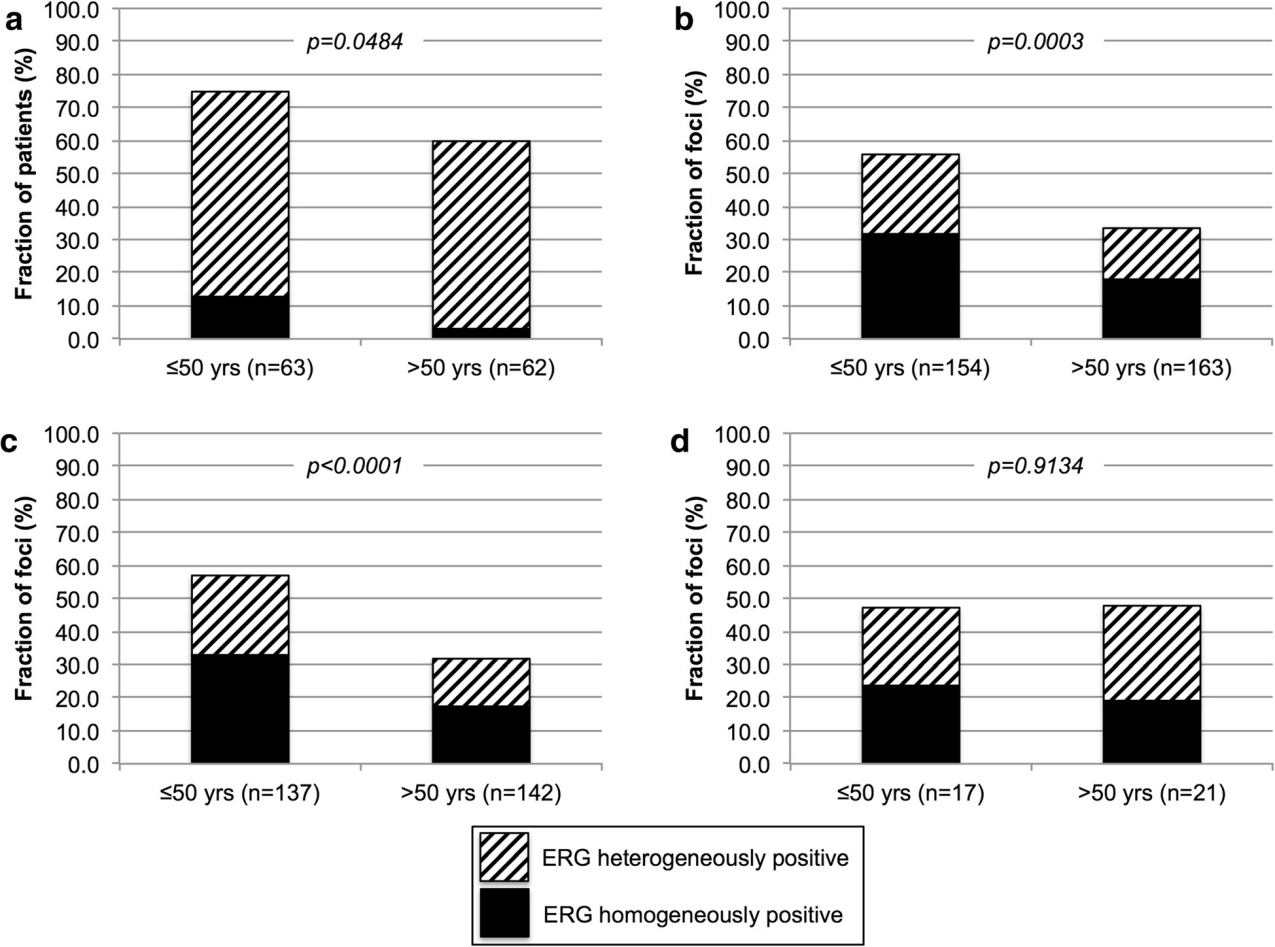
Association between patient age and the level of ERG heterogeneity on the basis of all 125 patients (**a**) and on a tumor focus basis in all 317 foci (**b**), as well as in the subsets of tumor foci with Gleason ≤3 + 4 (**c**) and ≥4 + 3 (**d**). Chi2 *p*-value was calculated across all groups (ERG homogenous negative, ERG homogenous positive and ERG heterogeneous positive)

In a recent study, we had demonstrated that positive ERG status is linked to young patient age [8]. The comparison of 63 EO-PCA (≤50 year) with 62 PCA of elderly patients (>50 year) showed again a difference in frequency of ERG positivity between these groups (Fig. 6a; *p* = 0.0484). This age difference became even more significant, if the analyses was done on a tumor focus level (Fig. 6b; *p* = 0.0003) and if the analysis was limited to low grade tumor foci with a Gleason ≤3 + 4 (Fig. 6c; *p* < 0.0001). An association of ERG status with patient age was not observed within 38 high-grade (Gleason ≥4 + 3) cancer foci (Fig. 6d; *p* = 0.9134).

### ERG expression in non-neoplastic prostate epithelia

**Fig. 7:**
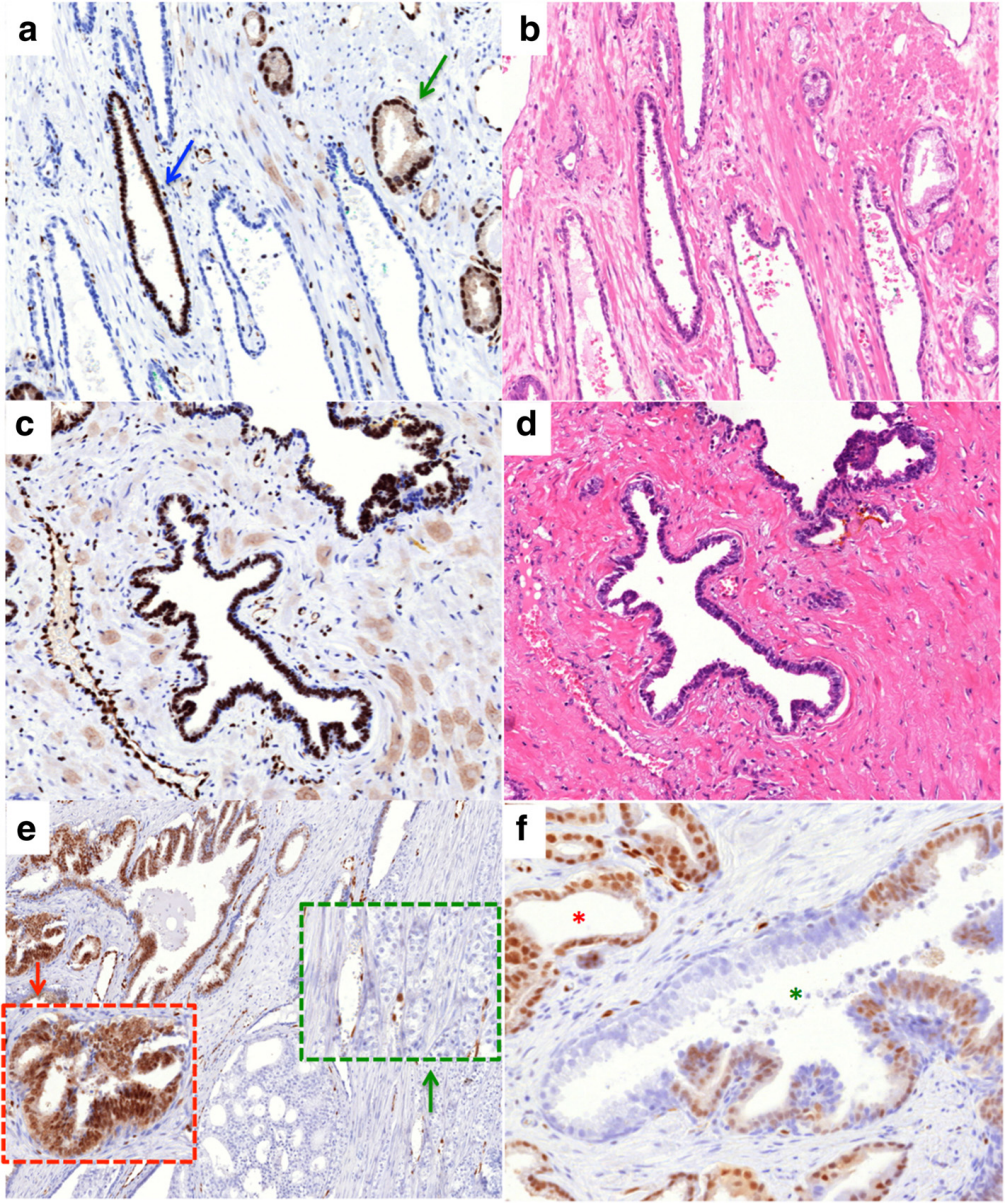
Representative images of ERG immunostainings. (**a**–**d**) Positive ERG immunostaining in non-neoplastic appearing prostate epithelium (**a** and **c**) with corresponding H&E staining (**b** and **d**). The *blue arrow* indicates normal prostate epithelium, the *green arrow* indicates cancer cells. (**e**) Positive staining in high-grade prostatic intraepithelial neoplasia (HGPIN, *red box*) and negative staining in prostate cancer (*green box*). (**f**) Heterogeneous ERG immunostaining in HGPIN (*green asterisk*). *Red asterisk* indicates invasive tumor cells

ERG staining was frequently found in high-grade prostatic intraepithelial neoplasia (HGPIN), where it always was heterogeneous. In ten tumor patients, ERG-positive small areas of non-neoplastic appearing prostatic epithelium were also seen. Examples of such findings are shown in Fig. 7.

## Discussion

The TMPRSS2:ERG fusion represents the most common genomic rearrangement in prostate cancer. Based on the pivotal effect of this fusion on prostate cancer cells by rendering ERG regulated genes androgen responsive, it was speculated that these fusions represent a major cancer initiating event [14]. Accordingly, it was proposed to distinguish “fusion-type” from “non fusion-type” prostate cancer as the two main molecular subtypes.

The data of this study suggest that pure “fusion-type” prostate cancer, where TMPRSS2:ERG fusions constitute a potential initiating event, may occur in not more than one third of all prostate cancer foci. This is based on our finding of homogeneous ERG positivity in 32 % of 59 small prostate cancer foci measuring 3 mm or less in diameter. That this percentage remains at comparable levels (14–36 %) irrespective of the tumor focus size is not surprising as cancers that were initially ERG positive are unlikely to loose TMPRSS2-ERG fusions during tumor progression.

The considerable fraction of 44.7 % heterogeneously ERG positive cancer foci and the continuous increase of ERG positive areas with tumor focus size found in our study further suggests that ERG fusion may not always be an initiating event but can also occur later during prostate cancer evolution. However, other studies reported less frequent intrafocal heterogeneity. For example, Barry et al. [11] found no unequivocal intrafocal heterogeneity in 32 multifocal prostate cancers, Furusato et al. [10] reported three tumors with signs of intrafocal heterogeneity in 81 multifocal cancers, Gumuskaya et al. [17] identified 7 % intrafocal heterogeneity in 44 ERG-positive tumor foci, Young et al. [16] found 4 % intrafocal heterogeneity in 78 ERG-positive tumor foci, and Svensson et al. [12] reported incidental intrafocal heterogeneity without specifying exact numbers. The markedly higher fraction of intrafocal ERG heterogeneity in unifocal cancers in our study is obviously due to the particularly large size of the majority of tumor foci (Fig. 1). It can be assumed, that the likelihood for subsequent ERG fusion development in initially ERG-negative cancer foci increases with tumor size and, therefore, over time. Alternatively, it cannot be excluded, that a certain fraction of unifocal cancers included in our study might represent “pseudo-unifocal tumors” resulting from collision of two or more independent tumor foci that cannot be distinguished histologically any more. However, given that individual tumor foci were defined according to generally accepted criteria in our study [21], that virtually all tumor foci showing potential intrafocal heterogeneity measured more than 4 mm, and that more than 80 % of the foci identified in our study measured >4 mm, our data suggests that either significant intrafocal heterogeneity exists, or that foci exeeding 4 mm are typically not unifocal even if they formally fulfill the criteria for unifocality.

The decreasing prevalence of completely ERG negative foci from 70 % to about 50 % with increasing tumor focus size suggest that subclones with TMPRSS2-ERG fusion develop in about 30 % of initially ERG negative cancer foci. The continuously high likelihood of prostate epithelial cells to develop TMPRSS2-ERG fusions (and other fusions linking ETS factors to androgen regulated genes) can be explained by the permanently activated androgen signaling in these cells. It has been shown that androgen signaling induces chromatin movements resulting in a close proximity of TMPRSS2 and ERG [22], including topological DNA constraints, which are resolved by topoisomerase 2B (TOP2B) mediated double strand breakage (DSB) and subsequent repair. Errors in this process result in recombinogenic TMPRSS2:ERG fusion and eventually in clonal selection of tumor cells carrying this alteration [23].

The large number of ERG stained sections that were carefully evaluated in the process of this study also lead to the identification of ten small areas of ERG-positive prostate epithelial cells that do not fulfill the morphologic criteria for cancer or high grade PIN. Although this observation may suggest that TMPRSS2-ERG fusions are not necessarily linked to malignancy, such rare findings may also be due to incidental false positive ERG IHC. Two previous studies suggested an error rate of 1:10,000 for ERG positivity based on similar rare ERG staining in benign epithelium [10, 16].

While pure “fusion-type” prostate cancer exists in up to 30 % on a tumor focus level, such a finding is an absolute rarity on the patient level, where homogeneous ERG positive cancers were only seen in ten patients (8 %). This finding was obviously caused by the high rate of interfocal heterogeneity in multifocal cancers. More than 60 % of our patients had more than one cancer focus in their prostates including 35 % with more than 3 cancer foci. While some of these cancers might have identical precursor lesions if they develop from one high grade PIN, it is apparent from our data, that most multifocal cancers represent independent “de novo” tumors since more than 60 % of multifocal cancers had both ERG positive and ERG negative foci. Presence of ERG positive and ERG negative subclones in the cancers of the vast majority of prostate cancer patients obviously challenges the classification of prostate cancers as “fusion-type” vs. “non-fusion type” on a patient level.

Based on our recent observation of a particularly high frequency of ERG fusions in early-onset prostate cancer we had hypothesized, that the development of ERG fusions is supported by the genuinely higher serum testosterone levels in younger than in older patients [8]. Based on the demonstrated impact of high testosterone levels facilitating ERG fusions in cell line models [22], it appears well possible, that the same mechanism may also apply in vivo. Our present data further validate the recently demonstrated association of ERG fusions with young patient age. Young patients not only have a higher likelihood to develop homogeneously ERG positive cancer foci (32 %) than old patients (18 %), they also have a higher likelihood for developing ERG positive subpopulations in initially ERG negative cancers.

It is a unique feature of our study, that a large series of cancers was assessed for heterogeneity by analyzing every individual cancer containing tissue block. The analysis involved a biomarker earlier considered a major classifier for prostate cancer. The very high rate of heterogeneity (89 %) found for “ERG positive” cancers highlights the importance of cancer heterogeneity. At times when drugs are increasingly administered based on the results of molecular analyses, and where drugs are being developed to target molecular features, it is of utmost importance to fully understand the impact of heterogeneity for potentially relevant molecular properties. It may be just by chance that Her2 - the most successful membranous drug target - is homogeneously expressed in >90 % of breast cancers, the main cancer type for anti-Her2 drugs. It is remarkable, that – at least in the literature - thorough heterogeneity analyses are still lacking for many drug targets under development.

## Conclusions

In summary, these data show, that homogeneous ERG positivity is very rare in prostate cancer, especially in elderly patients. However, development of subpopulations with ERG fusions may be a much more frequent event in ERG negative cancer foci as previously believed.

## Abbreviations

AR: Androgen Receptor
EOPCA: Early Onset Prostate Cancer
ERG: Erythroblast Transformation-Specific (Ets) Related Gene
IHC: Immunhistochemistry
TMPRSS2: Transmembrane Protease, Serine 2

## References

[1] Jemal A, Bray F, Center MM, Ferlay J, Ward E, Forman D (2011). Global cancer statistics. CA Cancer J Clin.

[2] Gulley J, Figg WD, Dahut WL (2003). Treatment options for androgen-independent prostate cancer. Clin Adv Hematol Oncol.

[3] Tomlins SA, Rhodes DR, Perner S, Dhanasekaran SM, Mehra R, Sun XW, Varambally S, Cao X, Tchinda J, Kuefer R (2005). Recurrent fusion of TMPRSS2 and ETS transcription factor genes in prostate cancer. Science.

[4] Park K, Tomlins SA, Mudaliar KM, Chiu YL, Esgueva R, Mehra R, Suleman K, Varambally S, Brenner JC, MacDonald T (2010). Antibody-based detection of ERG rearrangement-positive prostate cancer. Neoplasia.

[5] Minner S, Enodien M, Sirma H, Luebke AM, Krohn A, Mayer PS, Simon R, Tennstedt P, Muller J, Scholz L (2011). ERG status is unrelated to PSA recurrence in radically operated prostate cancer in the absence of antihormonal therapy. Clin Cancer Res.

[6] Attard G, Swennenhuis JF, Olmos D, Reid AH, Vickers E, A’Hern R, Levink R, Coumans F, Moreira J, Riisnaes R (2009). Characterization of ERG, AR and PTEN gene status in circulating tumor cells from patients with castration-resistant prostate cancer. Cancer Res.

[7] Karnes RJ, Cheville JC, Ida CM, Sebo TJ, Nair AA, Tang H, Munz JM, Kosari F, Vasmatzis G (2010). The ability of biomarkers to predict systemic progression in men with high-risk prostate cancer treated surgically is dependent on ERG status. Cancer Res.

[8] Weischenfeldt J, Simon R, Feuerbach L, Schlangen K, Weichenhan D, Minner S, Wuttig D, Warnatz HJ, Stehr H, Rausch T (2013). Integrative genomic analyses reveal androgen-driven somatic alteration landscape in early-onset prostate cancer. Cancer Cell.

[9] Minner S, Gartner M, Freudenthaler F, Bauer M, Kluth M, Salomon G, Heinzer H, Graefen M, Bokemeyer C, Simon R (2013). Marked heterogeneity of ERG expression in large primary prostate cancers. Mod Pathol.

[10] Furusato B, Tan SH, Young D, Dobi A, Sun C, Mohamed AA, Thangapazham R, Chen Y, McMaster G, Sreenath T (2010). ERG oncoprotein expression in prostate cancer: clonal progression of ERG-positive tumor cells and potential for ERG-based stratification. Prostate Cancer Prostatic Dis.

[11] Barry M, Perner S, Demichelis F, Rubin MA (2007). TMPRSS2-ERG fusion heterogeneity in multifocal prostate cancer: clinical and biologic implications. Urology.

[12] Svensson MA, LaFargue CJ, MacDonald TY, Pflueger D, Kitabayashi N, Santa-Cruz AM, Garsha KE, Sathyanarayana UG, Riley JP, Yun CS (2011). Testing mutual exclusivity of ETS rearranged prostate cancer. Lab Invest.

[13] Miyagi Y, Sasaki T, Fujinami K, Sano J, Senga Y, Miura T, Kameda Y, Sakuma Y, Nakamura Y, Harada M (2010). ETS family-associated gene fusions in Japanese prostate cancer: analysis of 194 radical prostatectomy samples. Mod Pathol.

[14] Furusato B, Gao CL, Ravindranath L, Chen Y, Cullen J, McLeod DG, Dobi A, Srivastava S, Petrovics G, Sesterhenn IA (2008). Mapping of TMPRSS2-ERG fusions in the context of multi-focal prostate cancer. Mod Pathol.

[15] Zhang S, Pavlovitz B, Tull J, Wang Y, Deng FM, Fuller C (2010). Detection of TMPRSS2 gene deletions and translocations in carcinoma, intraepithelial neoplasia, and normal epithelium of the prostate by direct fluorescence in situ hybridization. Diagn Mol Pathol.

[16] Young A, Palanisamy N, Siddiqui J, Wood DP, Wei JT, Chinnaiyan AM, Kunju LP, Tomlins SA (2012). Correlation of urine TMPRSS2:ERG and PCA3 to ERG+ and total prostate cancer burden. Am J Clin Pathol.

[17] Gumuskaya B, Gurel B, Fedor H, Tan HL, Weier CA, Hicks JL, Haffner MC, Lotan TL, De Marzo AM (2013). Assessing the order of critical alterations in prostate cancer development and progression by IHC: further evidence that PTEN loss occurs subsequent to ERG gene fusion. Prostate Cancer Prostatic Dis.

[18] Kononen J, Bubendorf L, Kallioniemi A, Barlund M, Schraml P, Leighton S, Torhorst J, Mihatsch MJ, Sauter G, Kallioniemi OP (1998). Tissue microarrays for high-throughput molecular profiling of tumor specimens. Nat Med.

[19] McNeal JE, Redwine EA, Freiha FS, Stamey TA (1988). Zonal distribution of prostatic adenocarcinoma. Correlation with histologic pattern and direction of spread. Am J Surg Pathol.

[20] Erbersdobler A, Hammerer P, Huland H, Henke RP (1997). Numerical chromosomal aberrations in transition-zone carcinomas of the prostate. J Urol.

[21] Wise AM, Stamey TA, McNeal JE, Clayton JL (2002). Morphologic and clinical significance of multifocal prostate cancers in radical prostatectomy specimens. Urology.

[22] Mani RS, Tomlins SA, Callahan K, Ghosh A, Nyati MK, Varambally S, Palanisamy N, Chinnaiyan AM (2009). Induced chromosomal proximity and gene fusions in prostate cancer. Science.

[23] Haffner MC, Aryee MJ, Toubaji A, Esopi DM, Albadine R, Gurel B, Isaacs WB, Bova GS, Liu W, Xu J (2010). Androgen-induced TOP2B-mediated double-strand breaks and prostate cancer gene rearrangements. Nat Genet.

